# Superspreading SARS Events, Beijing, 2003

**DOI:** 10.3201/eid1002.030732

**Published:** 2004-02

**Authors:** Zhuang Shen, Fang Ning, Weigong Zhou, Xiong He, Changying Lin, Daniel P. Chin, Zonghan Zhu, Anne Schuchat

**Affiliations:** * Beijing Center for Disease Prevention and Control, Beijing, China; † World Health Organization, Beijing, China; ‡ Centers for Disease Control and Prevention, Atlanta, Georgia, USA; § Beijing Joint SARS Expert Group, Beijing, China

**Keywords:** SARS virus, disease outbreaks, nosocomial infection, disease transmission, risk factors, epidemiology, contact tracing

## Abstract

Superspreading events were pivotal in the global spread of severe acute respiratory syndrome (SARS). We investigated superspreading in one transmission chain early in Beijing’s epidemic. Superspreading was defined as transmission of SARS to at least eight contacts. An index patient with onset of SARS 2 months after hospital admission was the source of four generations of transmission to 76 case-patients, including 12 healthcare workers and several hospital visitors. Four (5%) case circumstances met the superspreading definition. Superspreading appeared to be associated with older age (mean 56 vs. 44 years), case fatality (75% vs. 16%, p = 0.02, Fisher exact test), number of close contacts (36 vs. 0.37) and attack rate among close contacts (43% vs. 18.5%, p < 0.025). Delayed recognition of SARS in a hospitalized patient permitted transmission to patients, visitors, and healthcare workers. Older age and number of contacts merit investigation in future studies of superspreading.

One of the most intriguing aspects of coronavirus-associated severe acute respiratory syndrome (SARS) has been the circumstances under which virus is transmitted to large numbers of persons. One so-called superspreading event occurred in a Hong Kong hotel, when transmission from an ill traveler from Guangdong led to export of the virus to several other countries (*1*). Another highly effective episode of viral transmission occurred onboard China Air’s flight 112 from Hong Kong to Beijing on March 15, 2003 (*2*). Superspreading also played major roles in transmission of SARS within Singapore (*3*) and Toronto (*4*). The potential to transmit SARS-associated coronavirus (SARS-CoV) to large numbers of contacts is likely influenced by factors associated with the host, agent, and environment. To develop hypotheses for future international evaluation of this issue, reviewing the circumstances of transmission associated with individual superspreading events may be useful.

Beijing experienced the largest outbreak of SARS, with >2,500 cases reported between March and June 2003 (*2*). Several instances of superspreading were recognized during the Beijing epidemic, including two associated with imported cases, from Guangdong and Hong Kong, that each proved critical to the rapid increase in cases (*2*). Epidemiologic investigation of another chain of transmission that occurred early in Beijing’s outbreak permitted identification of several persons who spread SARS-CoV to many others. We describe this chain of transmission and the characteristics of superspreading detected in the course of its investigation.

## Methods

### Reporting

Potential cases of infectious atypical pneumonia, later called SARS, were reported by hospitals to the Beijing Center for Disease Control, which initiated epidemiologic investigations. Data sources included case report forms, epidemiologic investigation forms, and other investigation records at Beijing’s Center for Disease Control.

### Definitions

Cases were defined, in accordance with the “National Case Definition of Infectious Atypical Pneumonia (SARS) in China, 2003,” which was updated by the China Ministry of Health on April 23, 2003. Criteria for probable and suspected SARS included travel to a SARS-epidemic area in the 2 weeks before onset of symptoms or close contact with a probable SARS patient; fever of >38°C; chest x-ray abnormalities; normal or decreased leukocyte count; and no response to treatment with antimicrobial drugs.

Close contacts were identified according to the “Regulation of Beijing SARS close contact isolation, quarantine, service and supply.” The definition involved persons who shared meals, utensils, place of residence, a hospital room, or a transportation vehicle with a known probable or suspected SARS patient or had visited a SARS patient in a period beginning14 days before the patient’s onset of symptoms. Healthcare workers who examined or treated a SARS patient or any person who had potential contact with bodily secretions were also considered close contacts. We arbitrarily defined superspreading to occur when one SARS patient was attributed as the source of SARS in >8 other persons.

### Epidemiologic Investigation

We investigated probable and suspected cases reported from hospitals in Beijing to understand their relationship to each other, determine the incubation period between exposure and symptom onset, and describe clinical features at the time of symptom onset. We identified and followed close contacts of SARS patients to monitor their progress. We sought clinical data for patients associated with superspreading. The chi-square statistic and where appropriate, Fisher exact test, were used to compare proportions.

## Results

### Initial Infection and Transmission

**Figure 1:**
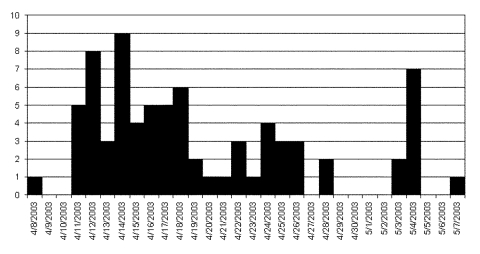
Epidemic curve of probable cases of severe acute respiratory syndrome, by date of onset of illness in one chain of transmission, Beijing 2003.

A 62-year-old woman (patient A)was admitted to a specialty hospital in Beijing for treatment of diabetes mellitus on February 5, 2003. The hospital treated a SARS patient in late March 2003, but specific contacts between that patient and patient A have not been identified. On April 5, 2003, fever and headache developed in patient A. Her leukocyte count was 6.4 x10^9^/L, and chest x-ray showed bilateral infiltrates with pleural effusion. She was treated for possible tuberculosis. Her clinical condition deteriorated, and she died April 12. On the same day, fever and chest x-ray abnormalities developed in eight of her relatives, including her husband, sons, daughters, and son-in-law, and they were diagnosed as having probable SARS (Figure 1).

**Figure 2:**
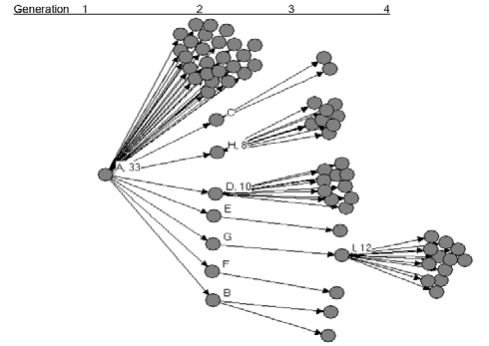
Probable cases of severe acute respiratory syndrome by source of transmission in chain of 77 cases in Beijing, 2003.

Patient A had 74 close contacts, including 25 healthcare workers, 11 relatives, 36 patients who were hospitalized in the same ward, and 2 persons who were accompanying other patients on the same ward. Among the close contacts, SARS developed in 33 of 74, for a secondary infection rate of 45% (Figure 2).

### Infection and Transmission among Second-Generation Patients

The 33 second-generation patients had 98 close contacts; SARS developed in 31 (32%). Nine (27%) of the 33 second-generation patients transmitted SARS to one or more contact.

Patients B and C were in the same ward as patient A and were discharged from the hospital after patient A was diagnosed with SARS. Each of them transmitted SARS to two relatives after discharge. The secondary infection rate among their contacts was 50% (4/8).

Patients D, E, F, G, and H were also hospitalized in the same ward as patient A, for the treatment of other diseases. They remained in the hospital after patient A was diagnosed with SARS. They later caused infection among visitors and some persons who accompanied them during their hospital stay. This hospital had not implemented isolation and quarantine procedures for SARS during this period.

Patient D (associated with superspreading) is a 70- year-old woman whose symptoms developed on April 13. She had five close contacts among her relatives; SARS did not occur in any of them. On April 12, patient L was admitted to the hospital for head trauma and placed in the same room as patient D. Patient L had 15 relatives who made frequent visits to the room; SARS developed in 10 of these, presumably from contact with patient D in the shared room. Among patient L’s family visitors to the room, the attack rate was 66.7% (10/15). Among all the visitors to the room (for patients D and L), the attack rate was 50% (10/20).

Patient H (associated with superspreading) is a 69-year-old woman whose symptoms developed on April 11, including chest x-ray with bilateral infiltrates. SARS developed in 8 of her 11 close contacts (secondary infection rate 73%). The second-generation patients E, F, and G each had one close contact; SARS developed in all three contacts.

Three additional persons (patients J, K, and Q) had been accompanying patients on the ward; symptoms of SARS developed in these three persons in the period April 12–18. Two of these (patients J and K) transmitted SARS to three contacts each. The other 22 second-generation patients had 32 close contacts; none developed SARS.

### Infection and Transmission among Third-Generation Patients

The 31 third-generation patients had 54 close contacts. Patient I was the only one who transmitted to others. Patient I, a 23-year-old man who had close contact with patient G, had onset of symptoms on April 25; unilateral abnormalities became visible on chest x-ray during the course of his illness. He had 45 close contacts with whom he either worked or lived; SARS occurred in 12 of these. The secondary attack rate among contacts of patient I was 27%.

### Outcomes of Illness among Patients in Infection Chain

A total of 77 SARS patients were in this chain of transmission, including 15 who died (including index case-patient A), for a case-fatality ratio of 20%. Case fatality was similar between the second and third generations (7/31, or 23%, second-generation cases, vs. 6/33, or 18%, third-generation patients]**.** All deaths occurred among persons >40 years of age. Case-patients who died averaged 63 years of age (range 41 to 82); surviving patients averaged 40 years (range 17 to 80) (p < 0.001).

### Analysis of Epidemiology of Superspreading

**Figure 3:**
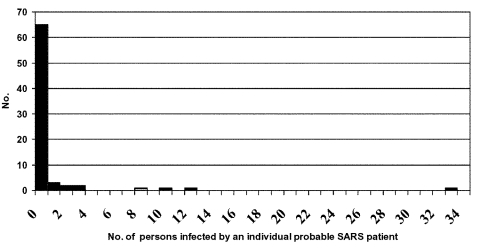
Number of direct secondary cases from probable cases of severe acute respiratory syndrome in one chain of transmission in Beijing, 2003.

Among the 77 patients, 66 did not transmit to others, and 7 transmitted to <3 contacts. In contrast, four persons (patients A, D, H, and I) transmitted to >8 others and were designated as associated with superspreading. The pattern of transmission is shown in Figure 3.

We compared the four case-patients associated with superspreading to the 73 other patients whose circumstances were associated with less frequent or no transmission. Patients linked to superspreading tended to be older than others in this transmission chain (mean 56 vs. 44.2 years) and a higher proportion were women (3/4 vs. 30/73, 41%, not significant by Fisher exact test). Three (75%) of four superspreaders died from their infection, compared with 12 (16%) of 73 others (p = 0.02, Fisher exact test, two tailed). Overall, healthcare workers accounted for 12 (16%) of the cases in this transmission chain, similar to the proportion of healthcare workers in the Beijing epidemic as a whole (16%) (*2*). None of the superspreading events involved transmission from healthcare workers.

We attempted a comparison of the number of close contacts of the index patient in superspreading events with the number of close contacts of other SARS patients; we also compared the proportion of close contacts in whom SARS developed for these two groups. Case-patients associated with superspreading averaged 36 contacts (range 11–74) while others averaged only 0.37 contacts. SARS developed in an average of 43% of close contacts of the four case-patients associated with superspreading; the syndrome developed in 18.5% of close contacts of the other patients. Thus superspreading appeared to be associated with a greater number of contacts and SARS developed in a higher proportion of those contacts (p < 0.025). These comparisons do not incorporate the susceptibility of contacts, but it is likely that the contacts of patient A represented a vulnerable population, since 36 (49%) of her 74 contacts were other hospitalized patients, while contacts of the later generation patients were primarily persons accompanying or visiting patients. Of note, five patients (B, C, E, F, G) who transmitted SARS to only 1–2 close contacts each had relatively few close contacts (range 1–4), which suggests limited opportunities for transmission instead of intrinsic differences in the transmissibility of their illness.

The epidemic curve for cases in this chain of transmission is shown in Figure 1. The three peaks of cases correspond to 1) second-generation patients, exposed to the index patient A (peak April 12–14), with a mean incubation period of 5.7 days; 2) third-generation patients (peak April 22–26); and 3) fourth-generation patients, peak May 4, all of whom had contact with patient I.

Cases clearly clustered in the hospital and within household members. The 77 cases involved 8 households and 1 construction site. There were 47 cases that represented secondary infection within households or workplaces, accounting for 61.3% of all patients. Seven of the eight families (77.8%) had more than two members with SARS. Sixty-two patients (81%) were either in the hospital before the onset of SARS or accompanied patients hospitalized on the same ward. Thus, even though there was transmission within most families, the place that family members were exposed in most of these cases was the hospital. Three of four superspreading events in this transmission chain occurred within the hospital; transmission from patient I was associated with a crowded construction site.

## Discussion

Our investigation highlights several features of SARS transmission observed in multiple outbreaks, including the central role of hospitals in disease transmission, the difficulty in distinguishing SARS from other clinical symptoms, and the danger associated with delayed case detection and isolation. Our investigation suggests that superspreading was related to both the environment (e.g., hospitals where large numbers of contacts occur) and host (patients who were older and had more severe illness). This transmission chain occurred relatively early in Beijing’s outbreak, and hospital authorities had not yet introduced personal protective equipment or isolation of patients with respiratory conditions.

The index patient in this report had been hospitalized for 2 months before clinical symptoms of SARS began. Early detection of SARS cannot simply focus on emergency room or outpatient encounters, since nosocomial infection may be the first indication of a cluster of illness. The patient’s condition was originally diagnosed as tuberculosis, another syndrome notable for potential for nosocomial transmission. Had they been implemented, appropriate respiratory precautions and patient isolation for suspected TB might have reduced hospital transmission of SARS. Improved infection-control standards for other conditions may benefit SARS control, and vice versa.

Transmission in three of the four superspreading events we describe occurred in the hospital setting. The hospital environment provided an efficient site for transmission, as was the case in other SARS outbreaks. Before administrative controls were introduced, our hospitalized patients had large numbers of contacts, including other patients, family members accompanying them during hospitalization, and other visitors. Other hospitalized patients are likely to be highly susceptible hosts because of older age and coexisting conditions. The viral load of hospitalized SARS patients is another potential factor; efficiency of SARS transmission increases in the 2nd week of illness, presumably as a function of viral load (*5*) or increasingly severe respiratory symptoms. The occurrence of SARS in many visitors to hospitals in Beijing and elsewhere highlights the need for administrative controls to restrict exposures to potentially infectious patients. Although not identified as factors in this transmission chain, certain aerosol-producing procedures, such as nebulizer treatments and emergency intubations, appeared to increase the risk for SARS transmission in other reports (*6*,*7*,*2*).

Superspreading appeared to be associated with patients who had larger numbers of close contacts as well as a higher attack rate among those contacts. These findings may be limited by bias introduced in assigning all patients hospitalized on the same ward to be contacts of the index patient. Although all case-patients were interviewed about close contacts, recall bias may have caused case-patients who were known to have transmitted to close contacts to be more thorough in identifying additional contacts. If we exclude patient A, the index patient, the average number of contacts for the three subsequent superspreading events was 24, with an attack rate among those contacts of 42%, still much higher than the corresponding numbers for other cases in this transmission chain (average 0.37 contacts and 18.5% attack rate). Although administrative controls instituted relatively late in this transmission chain reduced the number of contacts for some SARS patients, we cannot exclude the possibility that ascertainment of contacts for patients who did not transmit SARS was incomplete. In our investigation, the only example of superspreading outside the hospital setting occurred at a construction site; patient I had large numbers of contacts who worked and lived in crowded circumstances.

Superspreading was not associated with transmission from healthcare workers. Whether healthcare workers isolated themselves more promptly or had less opportunity for close contact is not known. Frequent handwashing by healthcare personnel might have contributed to lower rates of transmission. Because this outbreak occurred before personal protective equipment was routinely used, it is unlikely that use of masks or other such equipment was responsible for the low rate of transmission from healthcare workers to their contacts.

Our investigation raises hypotheses to be pursued in larger scale analysis of superspreading, such as whether demographic factors including female sex and older age are consistently associated with higher risk of transmitting to large numbers of others. Symptoms and signs evident upon illness onset should also be determined to identify clinical predictors of superspreading that might be integrated into triage protocols in the future. Additional features of the pathogen may also contribute to whether excessive transmission occurs, such as viral strain characteristics, viral load, or the presence of coinfecting organisms. Because most of the superspreaders we identified died from infection, the ability to gather additional information by retrospective interviews was limited. Future investigations will benefit from systematic and comprehensive prospective data collection from episodes of superspreading as well as comparison case circumstances.

SARS is not the only respiratory infection characterized by superspreading (*8*–*10*); other respiratory pathogens are often transmitted to large numbers of contacts. However, the severity of illness (i.e., radiographic pneumonia) attributable to SARS may make it easier to identify transmission chains and trace back to the index case in a given community. In contrast to influenza and outbreaks of most other respiratory infections, investigation of SARS outbreaks could usually uncover an index case. The impact that superspreading played on epidemics of SARS in individual outbreaks, as well as in transporting the virus between cities, underscores the need to recognize circumstances that facilitate widespread transmission so that control measures can be targeted appropriately. Thus, while superspreading is not unique to SARS, its occurrence in outbreaks may provide a guide to establishing critical points for disease control.

The global epidemiology of SARS in 2003 was greatly influenced by the occurrence of superspreading. Although numerous countries observed imported cases of SARS, few experienced local transmission. While some of the difference between the epidemiology of SARS after importation into different countries may be the result of preparedness and prompt patient isolation, the absence of a superspreading event was likely the dominant factor influencing which countries were spared epidemic spread. Pooling of information about superspreading may help shed additional light on the special set of circumstances required to disseminate infection to large numbers of contacts.

Before better predictors of superspreading are identified, triage procedures will require aggressive infection-control management of all possible SARS patients. After prompt measures were introduced in Beijing in response to the outbreak, opportunities for superspreading were greatly reduced. Thus there may have been many other patients with host or viral characteristics conducive to superspreading later in the Beijing outbreak, but successful infection control prevented these occurrences. As this transmission chain probably represents the natural history of SARS transmission before interventions were introduced, we can use these data to estimate the probability of superspreading in a given set of patients. Four (5%) of the 77 patients characterized in this transmission chain spread to >8 others. Thus, our data suggest that in the absence of interventions, superspreading is not a common event. However, the global experience with SARS in 2003 demonstrated that a single superspreading event can initiate a cascade of events that is difficult to interrupt. Improvement of laboratory assays to recognize SARS-CoV early in the clinical course may simplify infection-control strategies for patients with suspected SARS. However at present, clinical and epidemiologic characteristics are the only factors that are initially readily available to caregivers, and these must be scrutinized carefully to assure appropriate isolation procedures.
